# Integrating multi-marker phylogeny and morphology reveals hidden diversity and intraspecific morphological variation in the genus *Pseudohydnum* P. Karst. (*Basidiomycota*, *Auriculariales*) from China

**DOI:** 10.3897/mycokeys.142.215470

**Published:** 2026-10-28

**Authors:** Tai-Jie Yu, Wen-Hao Zhang, Xiu-Juan Li, Yuan-Yuan Xiong, Yu-Xian Gao, Ting Li, Rou Xue, Yuan-Tian Lu, Chang Xu, Jia Li, Li-Ping Tang

**Affiliations:** 1 School of Pharmaceutical Sciences and Yunnan Key Laboratory of Pharmacology for Natural Products, Kunming Medical University, Kunming, 650500, China School of Pharmaceutical Sciences and Yunnan Key Laboratory of Pharmacology for Natural Products, Kunming Medical University Kunming China; 2 Yunnan College of Modern Biomedical Industry, Kunming 650500, China Yunnan College of Modern Biomedical Industry Kunming China

**Keywords:** Bamboo host, edible mushrooms, jelly fungi, new species, taxonomy

## Abstract

The toothed jelly genus *Pseudohydnum* P. Karst. (*Basidiomycota*, *Auriculariales*) has historically been treated under a broad morphological concept centered on *P. gelatinosum*, a view that has long masked substantial cryptic diversity worldwide. However, many Chinese collections remain taxonomically unresolved and species boundaries are poorly defined. Here, we reassess the species diversity of Chinese *Pseudohydnum* through an integrative approach combining detailed morphology with multi-marker phylogenetics (ITS, nrLSU, and *rpb1*). We formally describe three new species, *P. ellipsosporum***sp. nov**., *P. macroumbrosum***sp. nov**., and *P. roseolostipitatum***sp. nov**. We also refine the circumscriptions of four previously described taxa and provide new insights into their intraspecific variation, geographic distribution, and ecology. Notably, we document a previously unknown host association: *P. brunneiceps* was consistently found on decayed bamboo (*Phyllostachys edulis*), a substrate never before recorded for any *Pseudohydnum* species. Critically, our results demonstrate that morphology alone is currently insufficient for species delimitation as convergent or plastic characters obscure diagnostic differences, reinforcing the necessity of molecular validation. These findings substantially expand the known taxonomic and ecological diversity of *Pseudohydnum* in Asia, suggesting that China, particularly the Qinghai-Xizang Plateau, may be a global hotspot for the genus. Overall, this study underscores the essential role of phylogenetic inference in revising species boundaries and elucidating evolutionary patterns in morphologically plastic groups of fungi.

## Introduction

The toothed jelly genus *Pseudohydnum* P. Karst. (*Basidiomycota*, *Auriculariales*) is widely distributed and is characterized by gelatinous, stipitate to sessile basidiomata, a hydnoid hymenophore, globose to ellipsoid basidiospores, and longitudinally septate basidia (Karsten 1868; Bessette et al. 1997; Weiß et al. 2004; Niveiro and Popoff 2011). Although initially placed in *Tremellales* based on basidial morphology (Karsten 1868; Breitenbach and Kränzlin 1986), the genus was reclassified to *Auriculariales* by Bandoni (1984), a placement subsequently confirmed by molecular phylogenetics, although its familial position within the order remains unresolved (Spirin et al. 2023; Coelho-Nascimento et al. 2024). In China, members are colloquially known as “cat’s tongue” (mao she jun) or “tiger’s paw” (hu zhang jun), and are valued both as edible mushrooms and as a source of bioactive compounds (Binion et al. 2008; Wu et al. 2019; Li et al. 2021; Sterniša et al. 2022).

Historically, the genus was dominated by its type species, *P. gelatinosum* (Scop.) P. Karst., which was regarded as a morphologically uniform and cosmopolitan taxon. Beyond its European type locality (Wojewoda 1981; Breitenbach and Kränzlin 1986; Pemberton 1994; Salo et al. 2005), the species has been widely reported from Asia (Hongo and Izawa 2006; Wu et al. 2009; Dai 2011; Li et al. 2015), North and South America (Lowy 1971; Courtecuisse and Lowy 1990; Volk et al. 1994; Mata et al. 2003; Binion et al. 2008; Niveiro and Popoff 2011), and Oceania (McNabb 1964; Fuhrer and Robinson 1992; Hood 1992; Ridley and Horne 2006; Gates and Ratkowsky 2016). Nevertheless, early mycologists such as Bourdot and Galzin (1927) already noted morphological variation among collections, especially from tropical regions, hinting at overlooked diversity. The advent of molecular tools has fundamentally reshaped our understanding, revealing that *P. gelatinosumsensu lato* represents a complex of phylogenetically distinct lineages rather than a single widespread species.

Recent systematic revisions integrating multi-marker DNA data with detailed morphology have significantly expanded the known diversity of *Pseudohydnum*. Spirin et al. (2023) revised *P. gelatinosum* and allies, re-established *Tremellodon pusillus* Ellis & Everh., and described six new species from Eurasia and North America. Concurrently, Coelho-Nascimento et al. (2024) unveiled four new Neotropical species from Brazil, highlighting that the diversity of the genus in tropical and subtropical ecosystems is substantially underestimated and geographically structured.

In China, taxonomic progress has accelerated markedly in recent years. Following the description of *P. brunneiceps*, the first modern species from the country by Chen et al. (2020), subsequent studies by Zhou et al. (2022, 2023) added five species, including *P. abietinum* H.M. Zhou & Jing Si, *P. candidissimum* H.M. Zhou, T. Bau & Jing Si (later synonymized with *P. translucens* Lloyd), *P. himalayanum* Y.C. Dai, F. Wu & H.M. Zhou, *P. sinobisporum* T. Bau, H.M. Zhou & Jing Si, and *P. sinogelatinosum* Y.C. Dai, F. Wu & H.M. Zhou. Intensive surveys on the Qinghai-Xizang Plateau further revealed four new species, *P. laricicola* Zhu L. Yang, Q. Cai & B. Feng, *P. motuoense* L. Lei & Q. Zhao, *P. purum* L. Lei & Q. Zhao, and *P. sororium* Zhu L. Yang & Q. Cai, alongside the first national records of *P. meridianum* Malysheva & Spirin and *P. cystidiatum* Malysheva & V. Dudka (Lei et al. 2025; Wang et al. 2025). Most recently, Zhang et al. (2026) and Guan et al. (2026) added *P. fusiforme* Xin Zhang & Y.C. Dai, *P. incarnatum* F. Wu, A. Tohtirjap, H.M. Zhou & Y.C. Dai, and *P. rhododendri* Xin Zhang & Y.C. Dai from Guangxi and Xizang, together with the first Chinese record of *P. umbrosum* V. Malysheva & Spirin in China. To date, the genus comprises 28 widely accepted taxa (27 species and one subspecies), with China harboring the majority of the known global diversity.

Despite these advances, the taxonomic inventory of *Pseudohydnum* in China remains incomplete. Many historical records from the country are based solely on morphology and likely encompass cryptic species. Furthermore, the phylogenetic relationships among Chinese taxa and their connections to extra-continental lineages are not yet fully resolved, and intraspecific morphological variation is insufficiently documented. The integration of comprehensive sampling, detailed morphological analysis, and multi-marker phylogenetics is therefore essential for delineating species boundaries and understanding the evolutionary history of the genus.

Here, we present an integrative taxonomic revision of *Pseudohydnum* in China, combining morphological and phylogenetic evidence. We formally describe three new species, reassess and refine four previously recognized taxa, and identify two additional putative lineages that require further collections for formal establishment. These findings substantially expand the known diversity of *Pseudohydnum* in China and highlight the urgent need for continued field exploration and molecular scrutiny of jelly fungi in underexplored regions, thereby contributing to a more refined understanding of the taxonomy and phylogeny of the genus.

## Materials and methods

### Collection sites and sampling

Fresh basidiomata were collected and photographed *in situ* from the Anhui, Xizang, and Yunnan provinces, China, between 2016 and 2025. Field data were recorded; a small portion of each collection was dried with silica gel for subsequent DNA extraction, while the remaining material was dried using an electric dehydrator at 40–55 °C and then stored in silica gel with a moisture indicator for long-term preservation. All voucher specimens are deposited in the Mycological Herbarium of Kunming Medical University (MHKMU) and the Herbarium Mycologicum Academiae Sinicae (HMAS).

### Morphological methods, notation, and standards

Macromorphological characters were described from field notes and *in-situ* photographs, with color notation following Kornerup and Wanscher (1978). For micromorphological examination, dried tissue sections were rehydrated in distilled water for 30–40 min, mounted in 5% KOH, lactic acid-based Cotton Blue, or Melzer’s reagent, and observed under a Leica DM2500 light microscope with bright-field illumination. In basidiospore descriptions, [*n/m/p*] indicates that *n* spores were measured from *m* basidiomata from *p* collections. Basidiospore dimensions are presented as (*a*–)*b*–*c*(–*d*), where the range *b*–*c* encompasses at least 90% of the measured values, and extreme values (*a* or *d*) are given in parentheses. The length/width ratio *Q* was calculated from spores in side view, and *Qm* represents the mean *Q* ± standard deviation. Basidiospore shapes were classified according to Bas (1969) and Yang (2005) based on *Q* values as follows: globose (1.0–1.05), subglobose (1.05–1.15), broadly ellipsoid (1.15–1.3), ellipsoid (1.3–1.6), elongate (1.6–2.0), cylindrical (2.0–3.0), and bacilliform (>3.0).

### DNA extraction, PCR amplification and sequencing

A total of 35 *Pseudohydnum* specimens were selected for molecular analysis. Genomic DNA was extracted and PCR amplification was performed following the protocols of Tang et al. (2014, 2017). The nuclear ribosomal internal transcribed spacer (ITS) region, the nuclear large subunit ribosomal DNA (nrLSU) gene, and the RNA polymerase II largest subunit (*rpb1*) gene were amplified using primer pairs ITS4/ITS5 (White et al. 1990), LR0R/LR5 (Vilgalys and Hester 1990), and RPB1-Af/RPB1-C2f (Matheny et al. 2002), respectively. PCR products were checked on 1% agarose gels and sequenced on an ABI 3730 DNA Analyzer with the same primers. Newly generated sequences were deposited on GenBank (Sayers et al. 2024).

### Alignment and molecular phylogenetic analyses

A total of 211 sequences (including 92 newly generated and 119 retrieved from GenBank), representing 92 collections, were included in the analyses (Table 1). Taxonomic authorities for all taxa and host species are provided in Suppl. material 1. Following previous studies, ingroup taxa comprised species closely related to our target species within *Pseudohydnum*. Two representatives of the genus *Stypellopsis* Spirin & Malysheva, *S. farlowii* (Burt) Spirin & K.H. Larss. and *S. hyperborea* Spirin & Malysheva were selected as outgroup taxa (Spirin et al. 2023; Zhang et al. 2026). The ITS, nrLSU, and *rpb1* datasets were individually aligned using MAFFT v7.310 (Katoh and Standley 2013) with the L-INS-i strategy, manually refined in BioEdit v7.0.9 (Hall 1999), and subsequently concatenated in PhyloSuite v1.2.3 (Zhang et al. 2020). Unsampled regions were coded as missing data.

**Table 1.** Specimen information and GenBank accession numbers of the sequences used in this study.

**Taxa**	**Voucher number**	**Locality**	**GenBank accession number**		
			**ITS**	**nrLSU**	***rpb1***
*Pseudohydnum abietinum* ^T^	Dai 24185	Gansu, China	OP965350	OP965370	—
*P. abietinum*	Dai 24194	Gansu, China	OP965351	OP965371	—
*P. alienum*	Kotiranta 22407	Finland	OM451494	OM451435	—
*P. alienum* ^T^	LE 253853	Russia	OM451493	OM451430	OM974204
*P. brasiliense*	MPD570	Brazil	OR625545	OR625561	PP805851
*P. brasiliense*	MPD536	Brazil	OR625543	OR625559	PP805850
*P. brunneiceps* ^T^	JXSB0967	Jiangxi, China	MN497254	MN497259	—
***P. brunneiceps***	**MHKMU LJ 140**	**Anhui, China**	**PZ671029**	**PZ671064**	**PZ859347**
***P. brunneiceps***	**MHKMU YTJ 129**	**Anhui, China**	**PZ671030**	**PZ671065**	**PZ859348**
***P. brunneiceps***	**MHKMU YTJ 129-1**	**Anhui, China**	**PZ671031**	**PZ671066**	**PZ859349**
*P. brunneovelutinum* ^T^	AGS118_2022	Brazil	OR625541	OR625557	PP805852
*P. brunneovelutinum*	NCC182	Brazil	OR625547	OR625563	PP805853
“*P. candidissimum*” ^T^	Dai 23740	Jilin, China	OP965345	OP965365	—
*P. cupulisnymphae*	NCC229	Brazil	OR625548	OR625564	PP805854
*P. cupulisnymphae* ^T^	NMJ420	Brazil	OR625556	OR625570	PP805855
*P. cystidiatum*	HKAS 137494	Yunnan, China	PQ871457	PV276931	—
*P. cystidiatum* ^T^	LE 313656	Vietnam	OM451503	OM451463	OM974207
***P. ellipsosporum***	**MHKMU YTJ 152**	**Yunnan, China**	**PZ671032**	**PZ671067**	—
***P. ellipsosporum* ^T^**	**MHKMU YTJ 198-1**	**Yunnan, China**	**PZ671033**	**PZ671068**	—
***P. ellipsosporum***	**MHKMU YTJ 198-2**	**Yunnan, China**	**PZ671034**	**PZ671069**	—
***P. ellipsosporum***	**MHKMU YTJ 198-3**	**Yunnan, China**	**PZ671035**	**PZ671070**	—
*P. fusiforme* ^T^	Dai 23556	Xizang, China	PZ043115	PZ043120	—
***P. fusiforme***	**HMAS272825**	**Xizang, China**	**PZ671036**	**PZ671071**	**PZ859350**
***P. fusiforme***	**HMAS272859**	**Xizang, China**	**PZ671037**	**PZ671072**	**PZ859351**
***P. fusiforme***	**MHKMU YTJ 405**	**Yunnan, China**	**PZ671038**	**PZ671073**	**PZ859352**
***P. fusiforme***	**MHKMU YTJ 424**	**Yunnan, China**	**PZ671039**	**PZ671074**	**PZ859353**
***P. fusiforme***	**MHKMU YTJ 425**	**Yunnan, China**	**PZ671040**	**PZ671075**	**PZ859354**
***P. fusiforme***	**MHKMU YTJ 426**	**Yunnan, China**	**PZ671041**	**PZ671076**	**PZ859355**
*P. gelatinosum* ^ET^	Spirin 13369	Slovenia	OM451472	OM451434	OM974193
***P. gelatinosum***	**HMAS262861**	**Italy**	**PZ671042**	**PZ671077**	—
“*P. gelatinosum*”	ANT187-QFB28623	Canada	MN992495	—	—
“*P. gelatinosum*”	ANT017-QFB28581	Canada	MN992496	—	—
*P. gelatinosum* subsp. *pusillum*	Miettinen 19625.1	USA	ON117834	ON117839	—
*P. gelatinosum* subsp. *pusillum*^NT^	Miettinen 18987.2	USA	ON117833	—	—
*P. himalayanum*	Cui 17035	Yunnan, China	ON243828	ON243926	—
*P. himalayanum* ^T^	Cui 17045	Yunnan, China	ON243829	ON243927	—
*P. incarnatum*	Wu791	Xizang, China	PP051393	PP051472	—
*P. laricicola* ^T^	HKAS 103433	Sichuan, China	PQ871449	PV650295	—
*P. laricicola*	HKAS 103450	Sichuan, China	PQ871450	PV276933	—
***P. laricicola***	**MHKMU HHY 897**	**Yunnan, China**	**PZ671043**	**PZ671078**	—
***P. laricicola***	**MHKMU YTJ 164**	**Yunnan, China**	**PZ671044**	**PZ671079**	—
***P. laricicola***	**MHKMU YTJ 168**	**Yunnan, China**	**PZ671045**	**PZ671080**	—
***P. laricicola***	**MHKMU YTJ 198**	**Yunnan, China**	**PZ671046**	**PZ671081**	**PZ859356**
***P. macroumbrosum* ^T^**	**MHKMU ZWH 537**	**Yunnan, China**	**PZ671047**	**PZ671082**	**PZ859357**
***P. macroumbrosum***	**MHKMU ZWH 537-1**	**Yunnan, China**	**PZ671048**	**PZ671083**	**PZ859358**
***P. macroumbrosum***	**MHKMU ZWH 661**	**Yunnan, China**	**PZ671049**	**PZ671084**	**PZ859359**
*P. meridianum* ^T^	LE 313568	Vietnam	OM451501	OM451452	—
*P. meridianum*	HKAS 145329	Yunnan, China	PV650322	PQ877882	—
*P. motuoense* ^T^	HKAS 134347	Xizang, China	PP999440	PP998493	—
*P. motuoense*	HKAS 134348	Xizang, China	PP999439	PP998492	—
*P. omnipavum*	Miettinen 18877	Canada	ON117836	OM451460	—
*P. omnipavum* ^T^	Spirin 8667	USA	OM451499	OM451441	OM974203
*P. orbiculare* ^T^	PDD 112653	New Zealand	ON243831	ON243929	—
*P. orbiculare*	PDD 112654	New Zealand	ON243832	—	—
*P. placibile* ^T^	LE 313658	Vietnam	OM451505	OM451465	OM974206
*P. placibile*	LE313659	Vietnam	OM451504	OM451462	OM974205
*P. purum* ^T^	HKAS 134352	Xizang, China	PP999437	PP998490	—
*P. purum*	HKAS 134353	Xizang, China	PP999438	PP998491	—
*P. rhododendri* ^T^	Cui 14495	Guangxi, China	PZ043112	PZ043118	—
*P. rhododendri*	Cui 14496	Guangxi, China	PZ043113	PZ043119	—
***P. roseolostipitatum***	**MHKMU LXJ 199**	**Yunnan, China**	**PZ671050**	**PZ671085**	**PZ859360**
***P. roseolostipitatum* ^T^**	**MHKMU YTJ 393**	**Yunnan, China**	**PZ671051**	**PZ671086**	**PZ859361**
***P. roseolostipitatum***	**HMAS277444**	**Yunnan, China**	**PZ671052**	**PZ671087**	**PZ859362**
*P. sinobisporum*	HMJAU 33728	Jilin, China	OP965349	OP965369	—
*P. sinobisporum* ^T^	SYL 2307	Jilin, China	OP965348	OP965368	—
***P. sinogelatinosum*** ^T^	Dai 23017	Yunnan, China	ON243836	ON243932	—
***P. sinogelatinosum***	**HMAS277542**	**Yunnan, China**	**PZ671053**	**PZ671088**	—
***P. sinogelatinosum***	**MHKMU NX 221**	**Yunnan, China**	**PZ671054**	**PZ671089**	—
***P. sinogelatinosum***	**MHKMU TLP 3439**	**Yunnan, China**	**PZ671055**	**PZ671090**	—
***P. sinogelatinosum***	**MHKMU YTJ 185**	**Yunnan, China**	**PZ671056**	**PZ671091**	—
***P. sinogelatinosum***	**MHKMU ZWH 647**	**Yunnan, China**	**PZ671057**	**PZ671092**	—
*P. sororium* ^T^	HKAS 108870	Yunnan, China	PQ871453	PQ877885	—
*P. sororium*	HKAS 145331	Yunnan, China	PQ871455	PQ877887	—
*P. tasmanicum* ^T^	Cui 16721	Australia	ON243838	ON243934	—
*P. tasmanicum*	Dai 18724	Australia	ON243839	ON243935	—
*P. totarae*	PDD 96246	New Zealand	ON243840	—	—
*P. totarae*	PDD 112655	New Zealand	ON243842	ON243936	—
*P. translucens*	LE313585	Russia	OM451497	OM451456	—
*P. umbrosum* ^T^	LE 312767	Russia	OM451500	OM451453	—
*P. viridimontanum* ^T^	NCC266	Brazil	OR625552	OR957373	—
*P. viridimontanum*	NCC273	Brazil	OR625553	OR957374	—
*Pseudohydnum* sp.	LJR 1213	Yunnan, China	PZ576836	—	—
*Pseudohydnum* sp.	LJR 1225	Yunnan, China	PZ576844	—	—
***Pseudohydnum* sp. 1**	**HMAS277560**	**Xizang, China**	**PZ671060**	**PZ671095**	**PZ859365**
***Pseudohydnum* sp. 1**	**HMAS275306**	**Xizang, China**	**PZ671061**	**PZ671096**	**PZ859366**
***Pseudohydnum* sp. 1**	**HMAS274300**	**Xizang, China**	**PZ671062**	**PZ671097**	**PZ859367**
***Pseudohydnum* sp. 1**	**HMAS275216**	**Xizang, China**	**PZ671063**	**PZ671098**	**PZ859368**
***Pseudohydnum* sp. 2**	**HMAS268332**	**Sichuan, China**	**PZ671058**	**PZ671093**	**PZ859363**
***Pseudohydnum* sp. 2**	**HMAS278655**	**Xizang, China**	**PZ671059**	**PZ671094**	**PZ859364**
*Pseudohydnum* sp. 3	iNaturalist#137211757	USA	PX609137	PX609278	—
*Stypellopsis farlowii*	GB:Larsson 12337	USA	MG857095	MG857099	—
*S. hyperborea*	Spirin 11066 (O)	Norway	MG857096	MG857102	—

**Note**: Bold = newly generated sequences in this study; — = sequence not available; T = holotype; NT = neotype; ET = epitype.

Phylogenetic trees were reconstructed using both maximum likelihood (ML) and Bayesian inference (BI) based on the concatenated ITS–nrLSU–*rpb1* dataset. The concatenated dataset was partitioned by genetic marker, and the best-fitting substitution models and optimal partitioning scheme were jointly selected using ModelFinder (Kalyaanamoorthy et al. 2017) with the MFP+MERGE option and --merge rcluster -rcluster 10 under the Bayesian information criterion (BIC). ML analyses were performed in IQ-TREE v3.1.2 (Wong et al. 2026) with 5,000 ultrafast bootstrap replicates (Minh et al. 2013; Hoang et al. 2018) and 1,000 replicates of the Shimodaira-Hasegawa approximate likelihood-ratio test (SH-aLRT) (Guindon et al. 2010), followed by bootstrap-based nearest-neighbor interchange optimization (--bnni). Two independent runs were conducted with a fixed random seed (--runs 2 --seed 12345) to ensure reproducibility. For BI, the same partitioned dataset was analyzed in MrBayes v3.2.7 (Ronquist et al. 2012) with the best-fit models selected by ModelFinder (ITS1: GTR+F+G4; 5.8S: F81+F; ITS2: HKY+F+G4; LSU: GTR+F+R3; *rpb1*: GTR+F+G4), running four independent MCMC chains for 2,000,000 generations, sampling every 100 generations. Convergence was verified by ensuring the average standard deviation of split frequencies remained below 0.01 and that effective sample sizes (ESS) exceeded 200. The first 25% of generations were discarded as burn-in, and Bayesian posterior probabilities (PP) were obtained from a majority-rule consensus tree constructed from the remaining trees.

## Results

### Phylogenetic analysis

**Figure 1.**
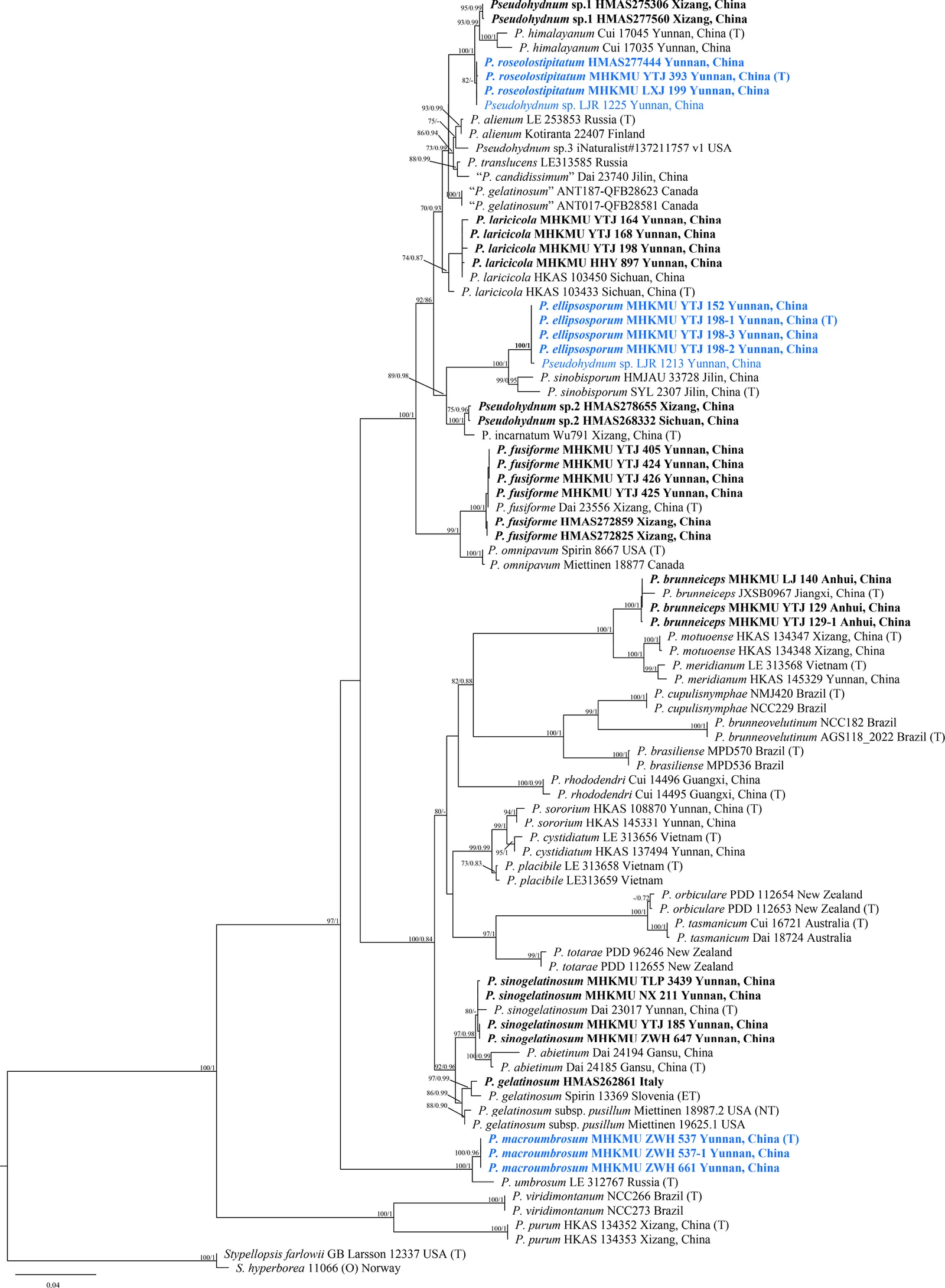
Bayesian phylogenetic tree of *Pseudohydnum* inferred from a concatenated dataset (ITS–nrLSU–*rpb1*). Bootstrap support values (ML) ≥ 70% and Bayesian posterior probabilities ≥ 0.80 are indicated on the branches. Sequences obtained from holotype, neotype, and epitype specimens are denoted by T, NT, and ET, respectively; newly generated sequences are in bold, and the new species described herein are highlighted in blue.

The concatenated ITS+nrLSU+*rpb1* dataset (https://doi.org/10.6084/m9.figshare.33684880) comprised 2,449 aligned nucleotide sites (including gaps): 669 from ITS, 893 from nrLSU, and 887 from *rpb1*. Bayesian inference (BI) and maximum likelihood (ML) analyses produced highly congruent and well-resolved topologies, with only minor differences in weakly supported nodes. The BI tree is presented in Fig. 1, with bootstrap support (BS) ≥ 70% and Bayesian posterior probabilities (PP) ≥ 0.80 indicated on the branches.

The multi-marker phylogeny unambiguously resolved the 35 newly collected specimens from China into nine strongly supported terminal clades. Four of these clades were assigned to known Chinese species: *P. brunneiceps*, *P. fusiforme*, *P. laricicola*, and *P. sinogelatinosum*. These assignments were based on both sequence identity and morphological concordance with their respective protologues. The remaining five clades could not be confidently matched with any described species; three of them are formally proposed as new species herein (*P. ellipsosporum* sp. nov., *P. macroumbrosum* sp. nov., and *P. roseolostipitatum* sp. nov.), while the other two (*Pseudohydnum* sp. 1 and sp. 2) are treated as putative new lineages awaiting more complete sampling.

Each of the three new taxa occupies strongly supported, phylogenetically distinct positions. *Pseudohydnum ellipsosporum* (BS = 100%, PP = 1.00) was recovered as sister to the northeastern Chinese *P. sinobisporum* (BS = 100%, PP = 1). *Pseudohydnum macroumbrosum* (BS = 100%, PP = 0.96) formed a well-supported sister clade to the far Eastern *P. umbrosum* Malysheva & Spirin (BS = 100%, PP = 1.00). *Pseudohydnum roseolostipitatum* (BS = 82) emerged as a distinct lineage sister to the Qinghai-Xizang plateau clade (BS = 100%, PP = 1) comprising *P. himalayanum* and an undescribed phylogenetic species.

Branch support values for the remaining clades were generally high, with most terminal clades receiving BS ≥ 95% and PP = 1.00, indicating robust phylogenetic placement of the newly described and previously known taxa. No significant conflict between ML and BI topologies was observed at well-supported nodes, suggesting stability of the inferred relationships.

### Taxonomy

**Kingdom *Fungi* Moore**

**Division *Basidiomycota* Moore**

**Class *Agaricomycetes* Doweld**

**Order *Auriculariales* Underw**.

**Genus *Pseudohydnum* P. Karst**.

#### Pseudohydnum brunneiceps

Taxon classificationFungiAuricularialesHyaloriaceae

Y.Lu Chen, M.S. Su & L.P. Zhang, Phytotaxa 441(1): 91 (2020)

72A0AD6B-BB19-5DEA-BC12-4934BB2CAA0C

Fig. 2

##### Description.

***Basidiomata*** medium-sized, gelatinous when fresh, pileate, laterally stipitate, solitary or gregarious. ***Pileus*** up to 40 mm in widest diameter and 6 mm thick, spathulate to flabellate or semicircular; pileal surface strigose, dark gray to grayish black, translucent when fresh; margin sharp to blunt, entire, undulating, fertile. ***Hymenophore*** hydnoid; spines sharp-tipped, 1–2 mm long, 2–3 per mm, milky white to pale grayish white. ***Stipe*** 30–40 mm long, 10–15 mm in diam., lateral, dark watery-grayish to gray, equal, compressed, translucent; surface strigose or partially covered with spines. ***Context*** gelatinous, translucent, unchanging. ***Odor and taste*** mild.

**Figure 2.**
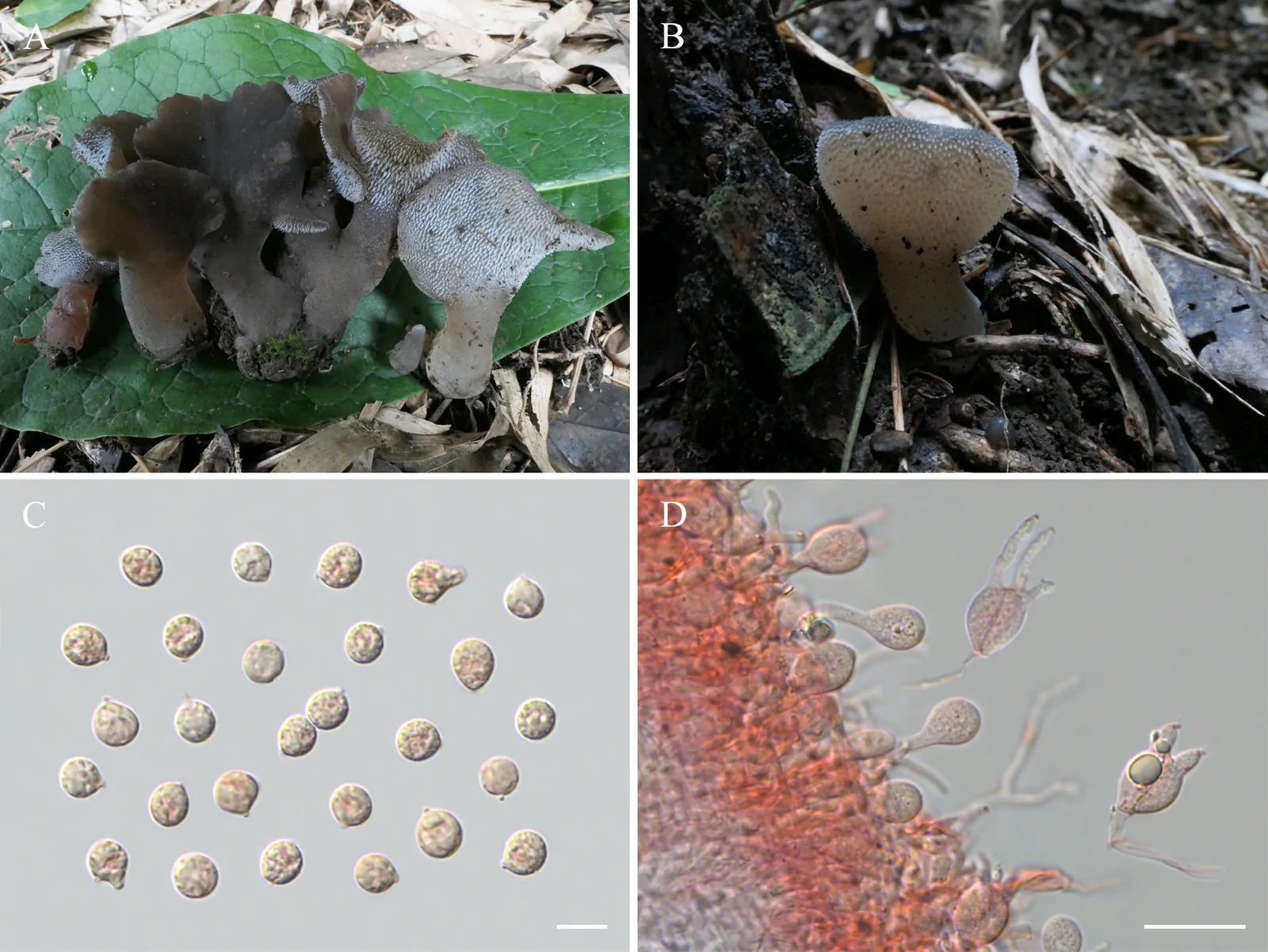
Basidiomata and microscopic structures of *Pseudohydnum brunneiceps*. **A, B**. Basidiomata *in situ* (**A** from MHKMU YTJ 129, **B** from MHKMU YTJ 129-1); **C**. Basidiospores; **D**. Basidia. Scale bars: 10 μm (**C**); 20 μm (**D**).

***Basidiospores*** [60/3/2] (7.2–)7.5–9.0(–9.5) × (6.7–)7.0–8.5(–8.8) μm, *Q* = (1.00–)1.02–1.15(–1.16), *Qm* = 1.07 ± 0.04, globose to subglobose, occasionally broadly ellipsoid, hyaline, smooth, thin-walled, nonamyloid, with a small apiculus, frequently germinating by repetition or by germ tubes on the surface. ***Basidia*** 11–16 × 8–11 μm, longitudinally cruciate-septate, predominantly 2-spored, occasionally 4-spored, arranged in a dense palisade extending to the spine apices; sterigmata up to 27 × 2–4 μm. ***Hyphidia*** 1–3 μm in diam., common, branched, with simple septa or clamp connections. ***Hyphal system*** monomitic, generative hyphae with clamp connections. ***Pileipellis*** composed of subparallel or ascending hyphae, 1–6 μm in diam., occasionally inflated up to 15 μm, thin- or slightly thick-walled, hyaline to brownish. ***Contextual hyphae*** 1−4 μm in diam., occasionally inflated up to 10 μm, interwoven, thin- to slightly thick-walled, hyaline. ***Subhymenial hyphae*** hyaline, thin-walled, interwoven or anastomosing, 2–7 μm in diam.

##### Ecology and distribution.

Growing on decayed conifer wood (predominantly *Cryptomeria japonica*) and *Phyllostachys edulis* (bamboo). Currently known from Central, East, and Southeast China, including Anhui, Hunan, and Jiangxi provinces.

##### Specimens examined.

China • Anhui Province, Shitai County (石台县), Xianyu Mountain Scenic Area (仙寓山风景区), 30°01'06"N, 117°16'34"E, elev. 1,050 m, on decaying *Phyllostachys edulis*, 21 July 2023, leg. T.J. Yu (MHKMU YTJ 129, MHKMU YTJ 129-1); • ibid., Dingxiang Town (丁香镇), 30°07'54"N, 117°19'26"E, elev. 370 m, on decaying *Phyllostachys edulis*, 18 July 2024, leg. J. Li (MHKMU LJ 140).

##### Comments.

*Pseudohydnum brunneiceps* was originally described from Jiangxi, China, on decayed coniferous wood, particularly *Cryptomeria japonica* (Chen et al. 2020), and later documented from Hunan on similar substrates (Spirin et al. 2023). Our collections extend the known distribution of *P. brunneiceps* to Anhui, where this species is locally common and harvested for consumption in the southern montane areas. Notably, contrary to previous reports of an exclusive conifer association (Chen et al. 2020; Spirin et al. 2023), all Anhui specimens were consistently found on decayed bamboo (*Phyllostachys edulis*), indicating a broader host range than previously recognized. To our knowledge, this is the first record of any *Pseudohydnum* species on bamboo.

#### Pseudohydnum ellipsosporum

Taxon classificationFungiAuricularialesHyaloriaceae

T.J. Yu & L.P. Tang sp. nov.

4EE56DF0-83AA-5D78-A972-E196BF5AFDE0

Fungal Names: FN 573833

Fig. 3

##### Chinese name.

椭圆孢刺银耳.

##### Diagnosis.

Similar to *P. laricicola*, but differs in its dark olive-brown pileus, compressed stipe, and longer and more elongate basidiospores (9.0–11.7 × 5.5–7.0 μm).

##### Holotype.

China • Yunnan Province, Ninglang County (宁蒗县), en route from Gewa Village (格瓦村) to Labo Town (拉伯乡), 27°43'23"N, 100°31'59"E, elev. 3,460 m, on decaying *Picea* wood, 2 October 2023, leg. T.J. Yu (***holotype***, MHKMU YTJ 198-1).

##### Etymology.

From Greek *elleipsis* (ellipse) and *spora* (spore), referring to the ellipsoid shape of the basidiospores, a key diagnostic feature of this species.

##### Description.

***Basidiomata*** large-sized, gelatinous when fresh, pileate, laterally stipitate to rarely subsessile, solitary. ***Pileus*** up to 55 mm in widest diameter and 10 mm thick, semicircular to flabellate; pileal surface strigose to pubescent, dark olive-brown (4E8) to dark brown (6E6), translucent when fresh; margin sharp, entire, undulated, upturned, fertile. ***Hymenophore*** hydnoid; spines sharp-tipped, up to 2 mm long, 2–3 per mm, white (1A1). ***Stipe*** 5–40 mm long, 6–24 mm in diam., lateral, white (1A1) to watery-grayish (1F1), compressed, translucent; surface densely covered by short spines, rudimentary in some specimens. ***Context*** gelatinous, translucent, unchanging. ***Odor and taste*** not recorded.

**Figure 3.**
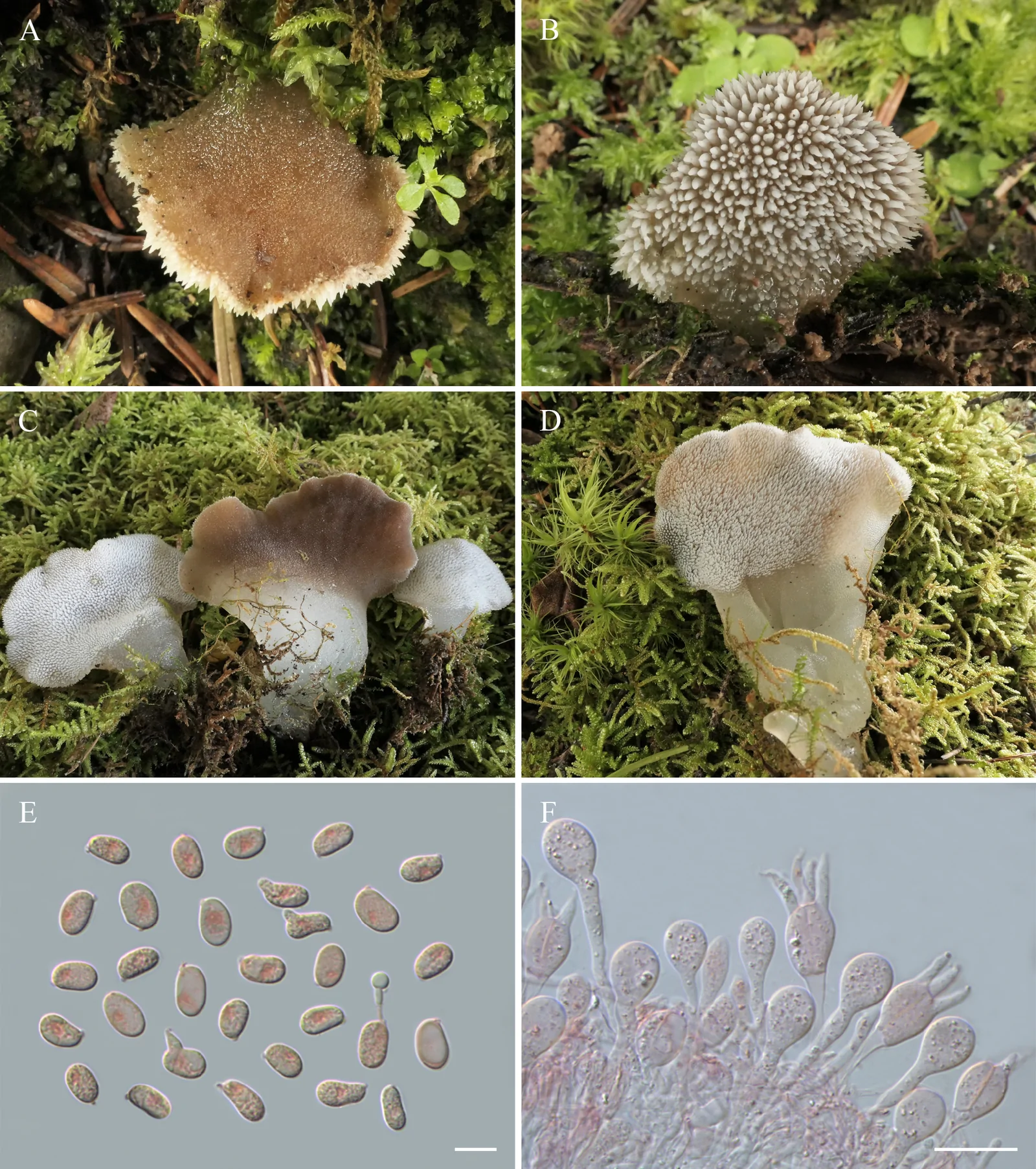
Basidiomata and microscopic structures of *Pseudohydnum ellipsosporum* sp. nov. (MHKMU YTJ 198-1, Holotype). **A–D**. Basidiomata *in situ* (**A, B** from MHKMU YTJ 152; **C, D** from MHKMU YTJ 198-1); **E**. Basidiospores; **F**. Basidia. Scale bars: 10 μm (**E**); 20 μm (**F**).

***Basidiospores*** [60/3/2] (8.7–)9.0–11.7(–12.0) × (4.5–)5.5–7.0(–7.2) μm, *Q* = (1.36–)1.46–2.11(–2.27), *Qm* = 1.71 ± 0.18, ellipsoid to elongate, occasionally cylindrical, hyaline, smooth, thin-walled, nonamyloid, with a small apiculus, frequently germinating by repetition or by germ tubes. ***Basidia*** 12–17 × 10–16 μm, longitudinally cruciate-septate, 4-spored, arranged in a dense palisade extending to the spine apices; sterigmata up to 17 × 2–4 μm. ***Hyphidia*** 1–3 μm in diam., common, branched, with simple septa or clamp connections. ***Hyphal system*** monomitic, generative hyphae with clamp connections. ***Pileipellis*** composed of subparallel or ascending hyphae, 1–7 μm in diam., occasionally branched and inflated up to 30 μm, thin- or slightly thick-walled, hyaline to brownish. ***Contextual hyphae*** 1−11 μm in diam., interwoven, thin- to slightly thick-walled, hyaline. ***Subhymenial hyphae*** hyaline, thin-walled, interwoven or anastomosing, 1–6 μm in diam., occasionally inflated up to 22 μm.

##### Ecology and distribution.

Growing on decayed conifer wood (*Picea* and *Taxus* spp.). Currently known only from Yunnan, southwestern China.

##### Other specimens examined.

China • Yunnan Province, Ninglang County (宁蒗县), en route from Gewa Village (格瓦村) to Labo Town (拉伯乡), 27°43'45"N, 100°32'02"E, elev. 3,260 m, on decaying *Picea* and *Taxus* wood, 30 September 2023, leg. T.J. Yu (***paratype***, MHKMU YTJ 152); • ibid., 27°43'23"N, 100°31'59"E, elev. 3,460 m, on decaying *Picea* wood, 2 October 2023, leg. T.J. Yu (MHKMU YTJ 198-2, MHKMU YTJ 198-3).

##### Comments.

*Pseudohydnum ellipsosporum* is distinguished by its large-sized, stipitate to subsessile basidiomata (pileus up to 55 mm in widest diameter), dark olive-brown pileus, and ellipsoid to elongate basidiospores (9.0–11.7 × 5.5–7.0 μm). Morphologically, it resembles *P. brunneovelutinum* C.C. Nascim. & Menolli and *P. meridianum*, but differs in basidiome size, stipe development, spine density, and spore dimensions. *Pseudohydnum brunneovelutinum* differs in its flabelliform to petaloid basidiomata with a short to rudimentary stipe, a smaller pileus (up to 35 mm in widest diameter), strongly and irregularly branched hyphidia, and its occurrence in tropical South America on angiosperm wood (Coelho-Nascimento et al. 2024). *Pseudohydnum meridianum* differs in its denser and shorter spines (0.5–1 mm long, 3–4 per mm), and smaller, subglobose to globose basidiospores (5.1–6.2 × 4.9–5.8 μm), and its occurrence on decayed wood in lowland evergreen mixed forest (Spirin et al. 2023; Wang et al. 2025).

Phylogenetically, *P. ellipsosporum* was recovered as sister to the temperate *P. sinobisporum* from Northeast China. However, the latter is readily distinguished by its smaller ivory pileus (up to 12 mm), shorter and more rounded basidiospores (7.5−9.5 × 5.8−7.2 μm), and predominantly 2-spored basidia (Zhou et al. 2023).

#### Pseudohydnum fusiforme

Taxon classificationFungiAuricularialesHyaloriaceae

Xin Zhang & Y.C. Dai, MycoKeys 131: 257 (2026)

37F2DA80-14B4-53B3-B046-34D67CD17A5C

Fig. 4

##### Description.

***Basidiomata*** small-sized, gelatinous when fresh, pileate, subsessile to laterally stipitate, solitary or gregarious (sometimes confluent at the base). ***Pileus*** up to 35 mm in widest diameter and 6 mm thick, spathulate, flabellate, semicircular, to shell-shaped; pileal surface minutely velutinous to pubescent, initially white to watery-grayish, transitioning through clay-pink or pale brown, and ultimately becoming brown, translucent when fresh; margin sharp to slightly blunt, entire, sometimes undulating, fertile. ***Hymenophore*** hydnoid; spines sharp-tipped, 0.8–1.5 mm long, 3–5 per mm, white. ***Stipe*** 5–10 mm long, 4–8 mm in diam., lateral, grayish white to watery-grayish, subequal, subcylindrical, translucent; surface often covered with fine spines, rudimentary in some specimens. ***Context*** gelatinous, translucent, unchanging. ***Odor and taste*** mild.

**Figure 4.**
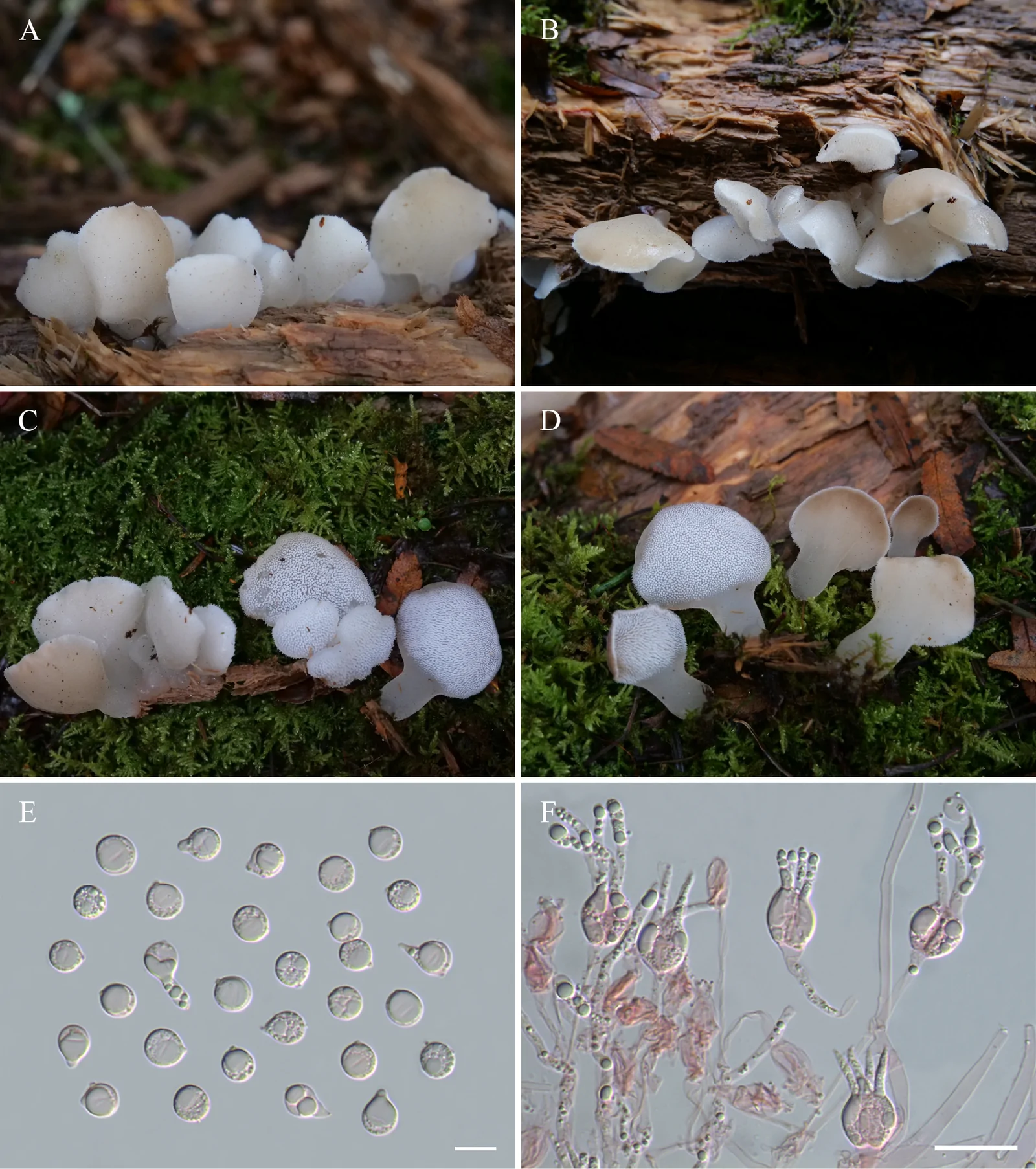
Basidiomata and microscopic structures of *Pseudohydnum fusiforme*. **A–D**. Basidiomata *in situ* (**A, B** from MHKMU YTJ 405; **C, D** from MHKMU YTJ 426); **E**. Basidiospores; **F**. Basidia. Scale bars: 10 μm (**E**); 20 μm (**F**).

***Basidiospores*** [60/3/3] (6.5–)7.0–8.8(–9.5) × (6.5–)6.8–8.5(–9.5) μm, *Q* = 1.00–1.09(–1.11), *Qm* = 1.04 ± 0.03, globose to subglobose, hyaline, smooth, thin-walled, nonamyloid, with a small apiculus, frequently germinating by repetition or by germ tubes. ***Basidia*** 11–14 × 10–13 μm, longitudinally cruciate-septate, 4-spored, arranged in a dense palisade extending to the spine apices; sterigmata up to 28 × 2–4 μm. ***Cystidia*** fusiform, 20–45 × 2–5 μm; ***Hyphidia*** 1–4 μm in diam., common, branched, with simple septa or clamp connections. ***Hyphal system*** monomitic, generative hyphae with clamp connections. ***Pileipellis*** composed of subparallel or ascending hyphae, 2–8 μm in diam., slightly inflated, thin- or slightly thick-walled, hyaline to brownish. ***Contextual hyphae*** 1−7 μm in diam., frequently branched, interwoven, thin- to slightly thick-walled, hyaline. ***Subhymenial hyphae*** hyaline, thin-walled, interwoven or anastomosing, 2–6 μm in diam., occasionally inflated up to 14 μm.

##### Ecology and distribution.

Growing on decayed coniferous wood (*Abies* spp.). Currently known only from Xizang and Yunnan, southwestern China.

##### Specimens examined.

China • Xizang Autonomous Region, Motuo County (墨脱县), near the Guoguotang Great Bend of the Yarlung Tsangpo River (雅鲁藏布江果果糖大拐弯), 29°18'N, 095°18'E, elev. 3,275 m, 18 September 2014, leg. W.L. Lu, X.D. Hu & Y.J. Yao (HMAS272859, HMAS272825); • Yunnan Province, Deqin County (德钦县), near Baima Snow Mountain pass (白马雪山), 28°24'30"N, 098°59'13"E, elev. 3,780 m, on decaying *Abies* wood, 4 October 2025, leg. T.J. Yu (MHKMU YTJ 405, MHKMU YTJ 424; MHKMU YTJ 425, MHKMU YTJ 426).

##### Comments.

*Pseudohydnum fusiforme* was recently described from Xizang, southwestern China, where it grows on fallen trunks of *Abies delavayi* var. *motuoensis* at high elevations (> 3,200 m) (Zhang et al. 2026). Here, we report this species for the first time from Yunnan Province. Our specimens conform to the diagnostic features (fusiform cystidia) but also exhibit notable morphological discrepancies from the protologue, including subsessile to laterally stipitate basidiomata, denser spines (3–5 per mm vs. 2 per mm); slightly wider and more globose basidiospores (7.0–8.8 × 6.8–8.5 μm vs. 7.0–8.5 × 5.5–7.0 μm), and a previously undocumented ontogenetic color change, progressing from white to watery-grayish, clay-pink, and ultimately brown. These findings expand both the known geographic range and the morphological circumscription of the species, suggesting that observed differences likely reflect intraspecific morphological variation.

Both *P. fusiforme* and *P. incarnatum* occur in Xizang on *Abies* and share fusiform cystidia, but they differ significantly in morphology. Macroscopically, *P. incarnatum* has a smaller, pink pileus (up to 23 mm vs. 35 mm wide) and sparser spines (2–3 vs. 3–5 per mm). Microscopically, *P. incarnatum* has wider cystidia (18.5–36.0 × 6.5–10.2 μm vs. 20–45 × 2–5 μm) and slightly narrower and more elongate basidiospores (6.0–8.2 × 5.0–6.2 μm vs. 7.0–8.8 × 6.8–8.5 μm) (Guan et al. 2026).

#### Pseudohydnum laricicola

Taxon classificationFungiAuricularialesHyaloriaceae

Zhu L. Yang, Q. Cai & B. Feng, Fungal Diversity 133: 309 (2025)

1179CEB0-F976-5870-BDB1-60E9421FAF96

Fig. 5

##### Description.

***Basidiomata*** medium to large-sized, gelatinous when fresh, pileate, laterally stipitate, solitary or gregarious. ***Pileus*** up to 50 mm in widest diameter and 5 mm thick, spathulate to flabellate or semicircular; pileal surface initially papillate and grayish white, becoming minutely velutinous to strigose and clay-pink to cinnamon, and finally brown at maturity, translucent when fresh; margin sharp to blunt, entire, undulating, fertile. ***Hymenophore*** hydnoid; spines sharp-tipped, 0.8–1.5 mm long, 2–3 per mm, white to milk white. ***Stipe*** 15–35 mm long, 5–15 mm in diam., lateral, watery-grayish to pale grayish white, equal, cylindrical, translucent; surface minutely velutinous to strigose or partially covered by spines. ***Context*** gelatinous, translucent, unchanging. ***Odor and taste*** mild.

**Figure 5.**
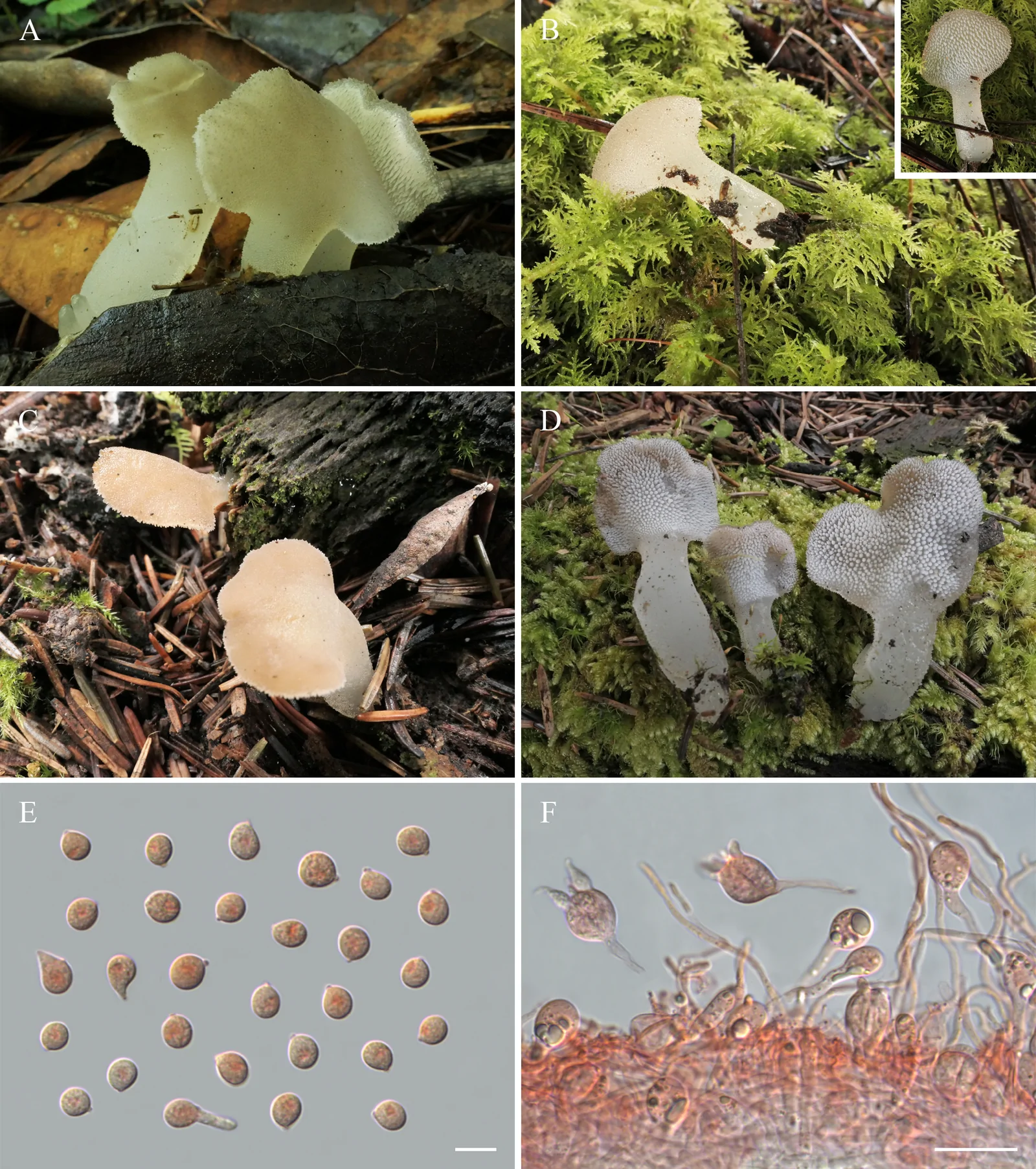
Basidiomata and microscopic structures of *Pseudohydnum laricicola*. **A–D**. Basidiomata *in situ* (**A** from MHKMU HHY 897; **B** from MHKMU YTJ 164; **C, D** from MHKMU YTJ 168); **E**. Basidiospores; **F**. Basidia. Scale bars: 10 μm (**E**); 20 μm (**F**).

***Basidiospores*** [60/3/3] (6.5–)6.8–8.3(–8.8) × (5.7–)6.2–7.5(–8.3) μm, *Q* = (1.02–)1.05–1.26(–1.30), *Qm* = 1.14 ± 0.06, subglobose to broadly ellipsoid, hyaline, smooth, thin-walled, nonamyloid, with a small apiculus, frequently germinating by repetition or by germ tubes. ***Basidia*** 11–14 × 10–13 μm, longitudinally cruciate-septate, predominantly 4-spored, occasionally 2-spored, arranged in a dense palisade extending to the spine apices; sterigmata up to 23 × 2–4 μm. ***Hyphidia*** 1–3 μm in diam., common, branched, with simple septa or clamp connections. ***Hyphal system*** monomitic, generative hyphae with clamp connections. ***Pileipellis*** composed of subparallel or ascending hyphae, 1–7 μm in diam., thin- or slightly thick-walled, hyaline to brownish. ***Contextual hyphae*** 1−4 μm in diam., interwoven, thin- to slightly thick-walled, hyaline. ***Subhymenial hyphae*** hyaline, thin-walled, interwoven or anastomosing, 1–7 μm in diam., occasionally inflated up to 15 μm.

##### Ecology and distribution.

Growing on decayed coniferous wood (predominantly *Larix*, *Picea*, and *Pinus*). Currently known only from Sichuan and Yunnan, southwestern China.

##### Specimens examined.

China • Yunnan Province, Ninglang County (宁蒗县), en route from Gewa Village (格瓦村) to Labo Town (拉伯乡), 27°43'42"N, 100°32'05"E, elev. 3,290 m, on decaying *Picea* wood, 30 September 2023, leg. T.J. Yu (MHKMU YTJ 164); • ibid., 27°43'25"N, 100°31'56"E, elev. 3,410 m, on decaying *Picea* wood, 1 October 2023, leg. T.J. Yu (MHKMU YTJ 168); • ibid., 27°43'23"N, 100°31'59"E, elev. 3,460 m, on decaying *Picea* wood, 2 October 2023, leg. T.J. Yu (MHKMU YTJ 198); • Shangri-la City (香格里拉市), near Yijia Village (彝族村), 27°23'04"N, 100°06'22"E, elev. 3,215 m, on decaying *Pinus armandii* wood, 30 July 2021, leg. H.Y. Huang (MHKMU HHY 897).

##### Comments.

*Pseudohydnum laricicola* was originally described from Sichuan, southwestern China, based on collections from decayed *Larix* wood (Wang et al. 2025). Our collections represent the first record from Yunnan, where the species appears relatively common on decayed wood of *Picea* and *Pinus armandii*. The basidiomata observed are notably larger than those in the original description (pileus up to 50 mm vs. 20–30 mm in diameter; stipe up to 35 mm vs. 10–20 mm long). Furthermore, pileus color varies distinctly with development—from initially watery-grayish to clay-pink/cinnamon and finally brown at maturity—and our basidiospores are slightly wider (6.8–8.3 × 6.2–7.5 μm vs. 5.5–7.5 × 5.5–6.5 μm) (Wang et al. 2025). These discrepancies likely stem from the original description having been based on immature or incompletely developed specimens; our findings significantly expand the known morphological range of this species.

#### Pseudohydnum macroumbrosum

Taxon classificationFungiAuricularialesHyaloriaceae

T.J. Yu & L.P. Tang sp. nov.

CF66F364-F659-5498-99CE-F327CA4F8D82

Fungal Names: FN 573834

Fig. 6

##### Chinese name.

大果暗色刺银耳.

##### Diagnosis.

Similar to and phylogenetically close to *P. umbrosum*, but differs in its significantly larger and thicker pileus, well-developed stipe, and more rounded basidiospores (8.3–10.0 × 7.3–8.7 μm).

##### Holotype.

China • Yunnan Province, Shangri-la City (香格里拉市), near the Yijia Village (彝族村), 27°23'07"N, 100°06'23"E, elev. 3,130 m, on decaying *Pinus armandii* wood, 26 August 2020, leg. W.H. Zhang (***holotype***, MHKMU ZWH 537).

##### Etymology.

From Greek *macro*- (large) and Latin *umbrosum* (dark-colored), referring to its phylogenetic and morphological affinity to *P. umbrosum* combined with its distinctly larger basidiomata.

**Figure 6.**
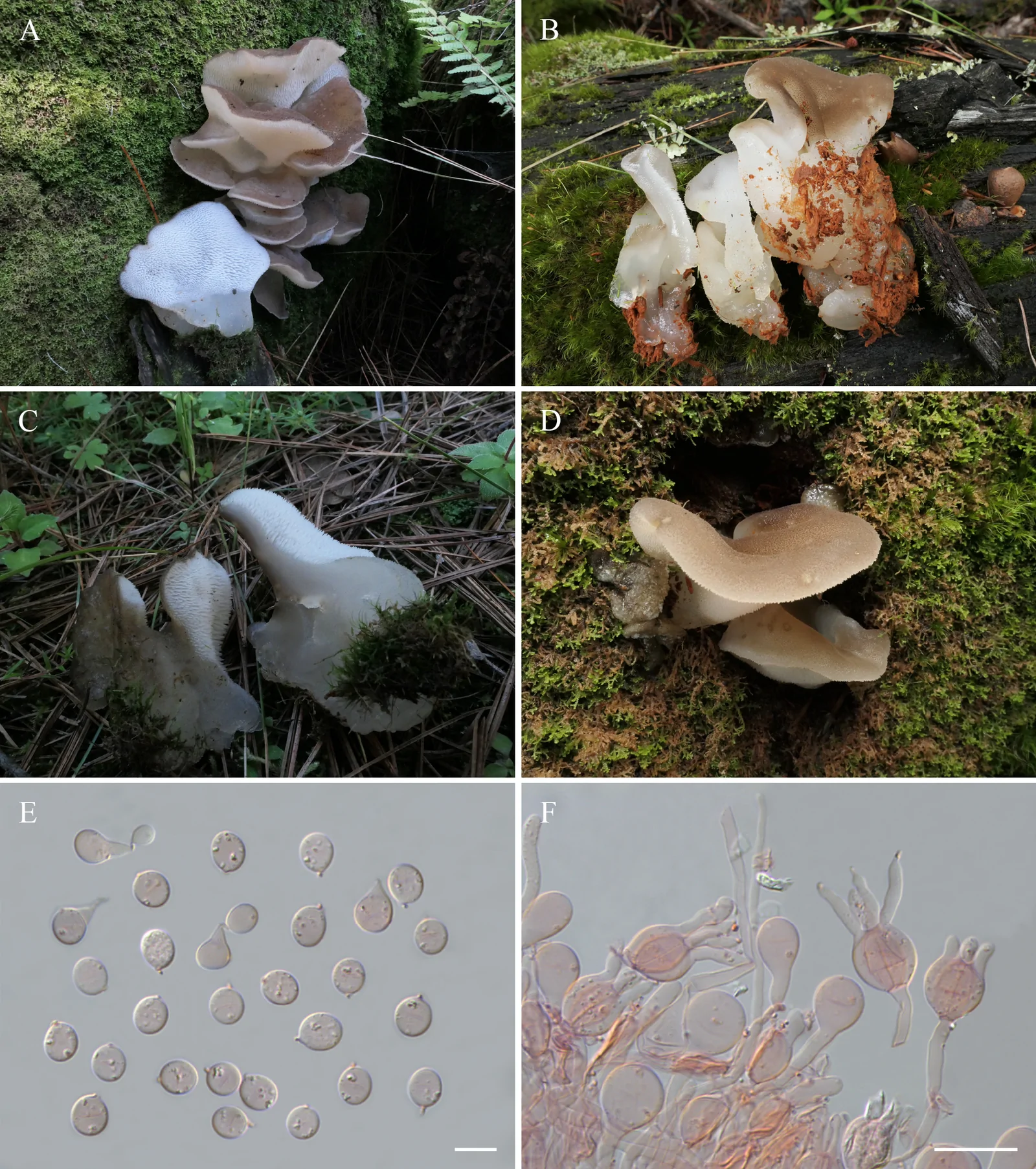
Basidiomata and microscopic structures of *Pseudohydnum macroumbrosum* sp. nov. (MHKMU ZWH 537, Holotype). **A–D**. Basidiomata *in situ* (**A, C** from MHKMU ZWH 537; **B, D** from MHKMU ZWH 661); **E**. Basidiospores; **F**. Basidia. Scale bars: 10 μm (**E**); 20 μm (**F**).

##### Description.

***Basidiomata*** large-sized, gelatinous when fresh, pileate, laterally stipitate, gregarious or imbricate (often confluent at the base). ***Pileus*** up to 110 mm in widest diameter and 10 mm thick, auriform to semicircular or flabellate; pileal surface papillate to minutely velutinous or strigose, pale brown (4B3) to pale yellowish brown (3B2) or olive brown (3D4), translucent when wet; margin sharp to slightly blunt, entire, rarely undulating, fertile. ***Hymenophore*** hydnoid; spines sharp-tipped, 1–3 mm long, 2–4 per mm, white (1A1). ***Stipe*** 30–60 mm long, 20–40 mm in diam., lateral, white (1A1) to watery-grayish (1B1), irregular, typically tapering upward from a bulbous base, somewhat compressed, translucent; surface covered with short spines. ***Context*** gelatinous, translucent, unchanging. ***Odor and taste*** mild.

***Basidiospores*** [60/2/2] (8.2–)8.3–10.0(–10.3) × (7.0–)7.3–8.7(–9.2) μm, *Q* = (1.02–)1.05–1.23(–1.25), *Qm* = 1.15 ± 0.05, subglobose to broadly ellipsoid, hyaline, smooth, thin-walled, nonamyloid, with a small apiculus, frequently germinating by repetition or by germ tubes. ***Basidia*** 13–16 × 10–16 μm, longitudinally cruciate-septate, 4-spored, arranged in a dense palisade extending to the spine apices; sterigmata up to 26 × 2–6 μm. ***Hyphidia*** 2–4.5 μm wide, common, branched, with simple septa or clamp connections. ***Hyphal system*** monomitic, generative hyphae with clamp connections. ***Pileipellis*** composed of subparallel or ascending hyphae, 2–8 μm in diam., slightly inflated, thin- or slightly thick-walled, hyaline to brownish. ***Contextual hyphae*** 2−9 μm in diam., frequently branched, interwoven, thin- to slightly thick-walled, hyaline. ***Subhymenial hyphae*** hyaline, thin-walled, interwoven or anastomosing, 2–7 μm in diam., occasionally inflated up to 17 μm.

##### Ecology and distribution.

Growing on decayed coniferous wood (*Pinus armandii*). Currently known only from Yunnan, southwestern China.

##### Other specimens examined.

China • Yunnan Province, Shangri-la City (香格里拉市), near Yijia Village (彝族村), 27°23'07"N, 100°06'23"E, elev. 3,130 m, on decaying *Pinus armandii* wood, 26 August 2020, leg. W.H. Zhang (MHKMU ZWH 537-1); • ibid., 27°23'10"N, 100°06'29"E, elev. 3,180 m, on decaying *Pinus armandii* wood, 30 July 2021, leg. W.H. Zhang (***paratype***, MHKMU ZWH 661).

##### Comments.

*Pseudohydnum macroumbrosum* is distinguished by its imbricate, often confluent basidiomata, exceptionally large pileus (up to 110 mm in widest diameter), well-developed stipe, and subglobose to broadly ellipsoid basidiospores (8.3–10.0 × 7.3–8.7 μm). Morphologically, it superficially resembles *P. brunneovelutinum*, *P. motuoense*, and *P. totarae* (Lloyd) J.A. Cooper, all of which have significantly smaller basidiomata and basidiospores. However, *P. brunneovelutinum* differs in its small-sized basidiomata with a short to rudimentary stipe base, sparse spines, smaller basidiospores (5.9–6.8 × 5.2–6.3 μm), and its occurrence on decayed angiosperm wood (Coelho-Nascimento et al. 2024). *Pseudohydnum motuoense* differs in its small basidiomata, slender stipe often expanding at the base, and much smaller basidiospores (5.5–6.8 × 4.6–6.0 μm) (Lei et al. 2025). *Pseudohydnum totarae* differs in having smaller, grayish brown to reddish brown pileus, smaller basidiospores (5.5–6.5 × 4.8–5.7 µm), and smaller basidia (Zhou et al. 2022).

Phylogenetically, *P. macroumbrosum* is sister to *P. umbrosum*, but the latter has nearly sessile basidiomata, a much smaller pileus (≤ 20 mm), and narrower basidiospores (7.6–9.8 × 6.0–7.1 μm) (Spirin et al. 2023).

#### Pseudohydnum roseolostipitatum

Taxon classificationFungiAuricularialesHyaloriaceae

T.J. Yu & L.P. Tang sp. nov.

17208462-C0CD-50CD-B4A0-1D3A6C92497B

Fungal Names: FN 573835

Fig. 7

##### Chinese name.

长柄粉盖刺银耳.

##### Diagnosis.

Similar to and phylogenetically close to *P. himalayanum*, but differs in its well-developed stipe often expanding at the base, pinkish gray to cinnamon brown pileus, slightly sparse spines (2–4 per mm), and globose to subglobose basidiospores and basidia.

##### Holotype.

China • Yunnan Province, Deqin County (德钦县), 28°24'36"N, 098°59'4"E, elev. 3,770 m, on decaying *Abies* wood, 3 October 2025, leg. T.J. Yu (***holotype***, MHKMU YTJ 393).

##### Etymology.

From Latin *roseolus* (pale pink) and *stipitatus* (stipe), referring to the pale pink pileus and well-developed stipe of this species.

##### Description.

***Basidiomata*** medium-sized, gelatinous when fresh, pileate, laterally stipitate, solitary or gregarious (rarely confluent at the base). ***Pileus*** up to 45 mm in widest diameter and 3 mm thick, spathulate to flabellate or semicircular; pileal surface minutely velutinous to smooth, pinkish gray (7B3) to cinnamon brown (6C5, 6D6), translucent when fresh; margin sharp to slightly blunt, entire, undulating, fertile. ***Hymenophore*** hydnoid; spines sharp-tipped, 1–2 mm long, 2–4 per mm, white (1A1). ***Stipe*** 30–40 mm long, 10–17 mm in diam., lateral, watery-grayish (1B1) to pale brownish gray (3B2), clavate, typically tapering upward from a swollen, sometimes bulbous base, translucent; surface generally smooth, rarely bearing spines. ***Context*** gelatinous, translucent, unchanging. ***Odor and taste*** mild.

**Figure 7.**
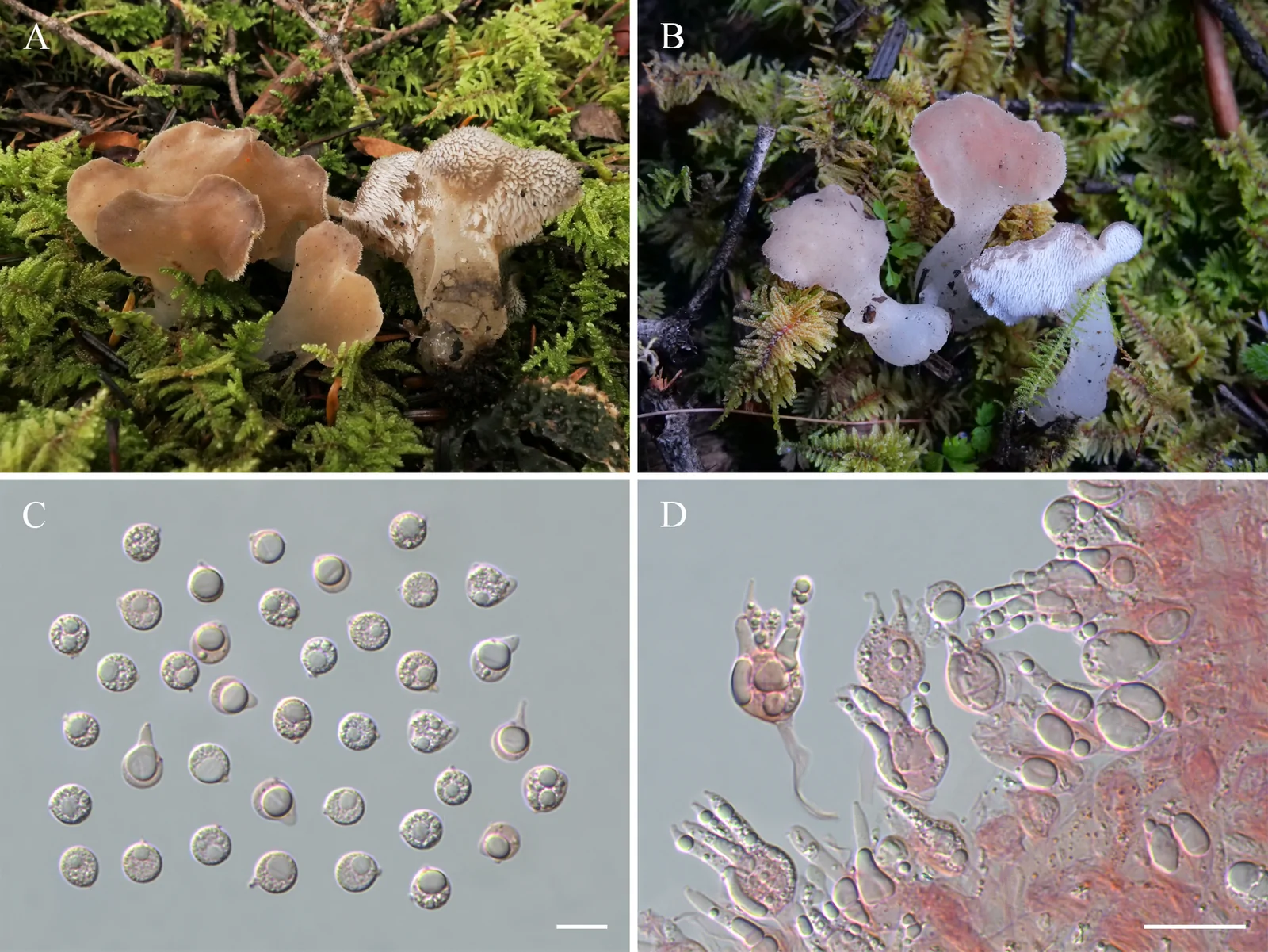
Basidiomata and microscopic structures of *Pseudohydnum roseolostipitatum* sp. nov. (MHKMU YTJ 393, Holotype). **A, B**. Basidiomata *in situ* (**A** from MHKMU LXJ 199; **B** from MHKMU YTJ 393); **C**. Basidiospores; **D**. Basidia. Scale bars: 10 μm (**C**); 20 μm (**D**).

***Basidiospores*** [60/3/3] (6.7–)7.0–8.3(–8.5) × (6.3–)6.5–7.8(–8.3) μm, *Q* = (1.00–)1.01–1.13(–1.17), *Qm* = 1.05 ± 0.04, globose to subglobose, occasionally broadly ellipsoid, hyaline, smooth, thin-walled, nonamyloid, with a small apiculus, frequently germinating by repetition or by germ tubes. ***Basidia*** 10–15 × 9–15 μm, longitudinally cruciate-septate, 4-spored, arranged in a dense palisade extending to the spine apices; sterigmata up to 15 × 2–4 μm. ***Hyphidia*** 1–4 μm in diam., common, branched, with simple septa or clamp connections. ***Hyphal system*** monomitic, generative hyphae with clamp connections. ***Pileipellis*** composed of subparallel or ascending hyphae, 1–6 μm in diam., slightly inflated, thin- or slightly thick-walled, hyaline to brownish. ***Contextual hyphae*** 2−12 μm in diam., interwoven, thin- to slightly thick-walled, hyaline. ***Subhymenial hyphae*** hyaline, thin-walled, interwoven or anastomosing, 2–9 μm in diam., occasionally inflated up to 18 μm.

##### Ecology and distribution.

Growing on decayed coniferous wood (*Abies* spp.). Currently known only from Yunnan, southwestern China.

##### Other specimens examined.

China • Yunnan Province, Deqin County (德钦县), Yunling Town (云岭乡), 28°21'29"N, 098°49'50"E, elev. 3,800 m, 11 August 2016, leg. Y. Li (HMAS277444); • ibid., near the Baima Snow Mountain pass, 28°24'20"N, 098°59'09"E, elev. 3,800 m, from decaying *Abies* wood, 4 October 2025, leg. X.J. Li (***paratype***, MHKMU LXJ 199).

##### Comments.

*Pseudohydnum roseolostipitatum* is distinguished by its medium-sized basidiomata, well-developed clavate stipe expanding at the base, pinkish gray to cinnamon brown pileus, and globose to subglobose basidiospores (7.0–8.3 × 6.5–7.8 μm). Among sympatric taxa, it differs from *P. himalayanum* (denser spines 5–6 per mm and broadly ellipsoid to subglobose basidiospores), *P. incarnatum* (short or sometimes rudimentary stipe, fusiform cystidia, and broadly ellipsoid to subglobose basidiospores) and *P. laricicola* (subcylindrical stipe lacking a bulbous base, more elongate basidiospores) (Zhou et al. 2022; Wang et al. 2025; Guan et al. 2026; this study).

For non-overlapping species with pinkish to cinnamon-brown pigmentation, such as *P. brasiliense* C.C. Nascim., Zabin & Menolli, *P. gelatinosum* subsp. *pusillum*, and *P. omnipavum*, further distinctions are noted. *Pseudohydnum brasiliense* differs in its pubescent pileus, significantly shorter and thinner stipe (6–11 × 3.5–6 mm), and predominantly 2-spored basidia (Coelho-Nascimento et al. 2024). *Pseudohydnum gelatinosum* subsp. *pusillum* is readily distinguished by its much shorter and thinner, or sometimes rudimentary, stipe (up to 5 mm long), which is typically covered with spines extending to the base (Spirin et al. 2023). *Pseudohydnum omnipavum* produces distinctly smaller basidiomata (up to 20 mm in diam.) with a much shorter and thinner stipe (up to 5 mm long). It is associated with *Pseudotsuga* and *Tsuga* (Spirin et al. 2023).

Phylogenetically, *P. roseolostipitatum* shows a close affiliation with the Qinghai-Xizang Plateau clade containing *P. himalayanum* Y.C. Dai, F. Wu & H.M. Zhou but occupies an independent, strongly supported branch.

#### Pseudohydnum sinogelatinosum

Taxon classificationFungiAuricularialesHyaloriaceae

Y.C. Dai, F. Wu & H.M. Zhou, J. Fungi 8 (7, no. 658): 7 (2022)

7625508A-B6E1-5B54-BD2E-98DF5889EA1A

Fig. 8

##### Description.

***Basidiomata*** large-sized, gelatinous when fresh, pileate, laterally stipitate, solitary or gregarious (often confluent at the base). ***Pileus*** up to 60 mm in widest diameter and 15 mm thick, flabellate or semicircular; pileal surface smooth to minutely velutinous or strigose, varying from pale grayish white, pale clay-pink to sorrel brown, translucent when fresh; margin blunt, entire, undulating, fertile. ***Hymenophore*** hydnoid; spines sharp-tipped, 1–5 mm long, 1–3 per mm, white to milk white. ***Stipe*** 10–25 mm long, 10–17 mm in diam., lateral, watery-grayish to pale grayish white, subcylindrical to slightly compressed, translucent; surface minutely velutinous to strigose or partially bearing spines. ***Context*** gelatinous, translucent, unchanging. ***Odor and taste*** mild.

**Figure 8.**
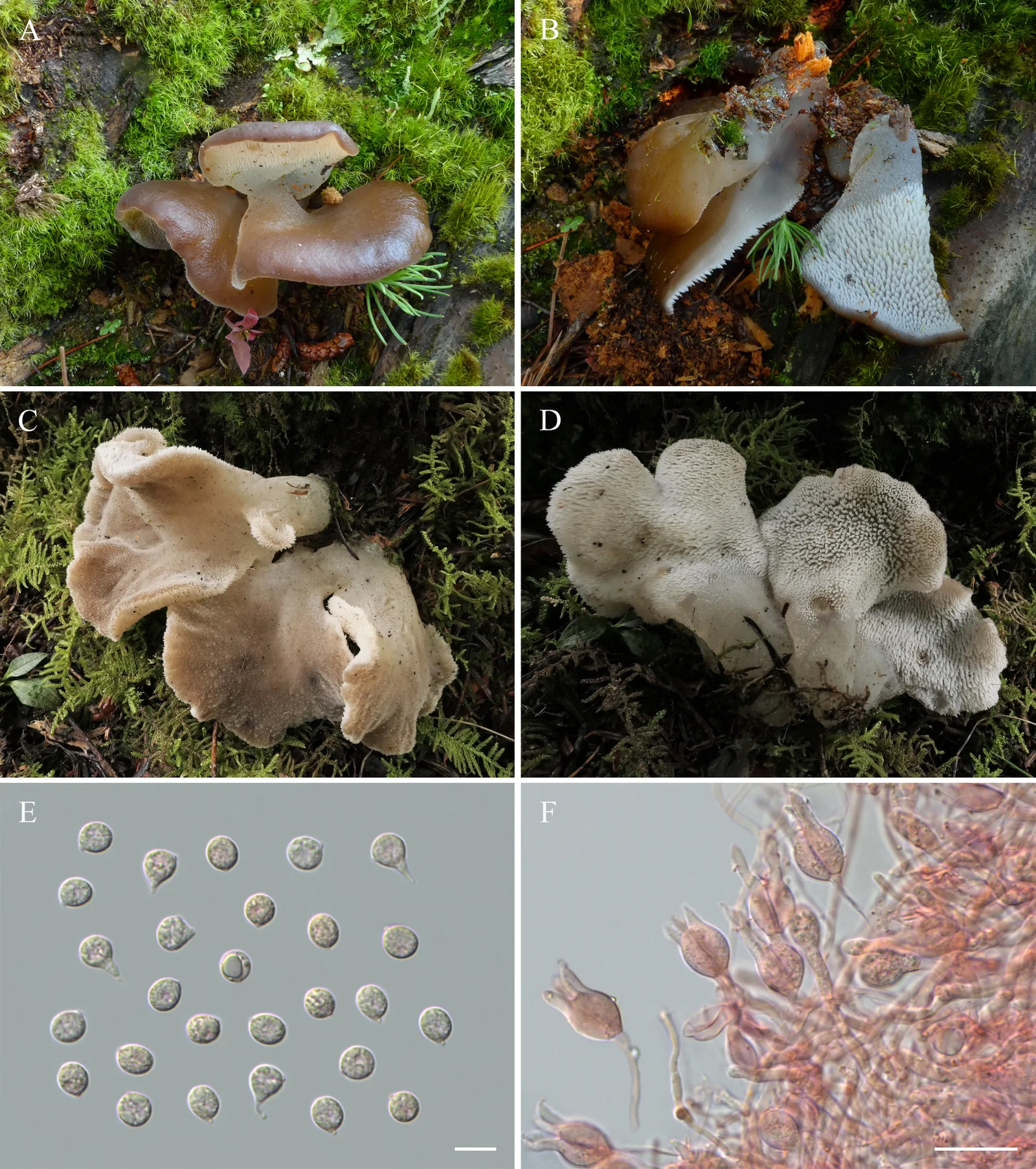
Basidiomata and microscopic structures of *Pseudohydnum sinogelatinosum*. **A–D**. Basidiomata *in situ* (**A, B** from MHKMU TLP 3439; **C, D** from MHKMU YTJ 185); **E**. Basidiospores; **F**. Basidia. Scale bars: 10 μm (**E**); 20 μm (**F**).

***Basidiospores*** [60/3/2] (7.0–)7.2–8.8(–9.2) × (6.2–)6.7–8.2(–8.5) μm, *Q* = (1.02–)1.03–1.24(–1.27), *Qm* = 1.11 ± 0.06, globose to broadly ellipsoid, hyaline, smooth, thin-walled, nonamyloid, with a small apiculus, frequently germinating by repetition or by germ tubes. ***Basidia*** 12–14 × 9–12 μm, longitudinally cruciate-septate, predominantly 4-spored, occasionally 2-spored, arranged in a dense palisade extending to the spine apices; sterigmata up to 14 × 2–4 μm. ***Hyphidia*** 1–3 μm in diam., common, branched, with simple septa or clamp connections. ***Hyphal system*** monomitic, generative hyphae with clamp connections. ***Pileipellis*** composed of subparallel or ascending hyphae, 1–4 μm in diam., occasionally inflated up to 11 μm, thin- or slightly thick-walled, hyaline to brownish. ***Contextual hyphae*** 1−7 μm in diam., rarely inflated up to 14 μm in diam., interwoven, thin- to slightly thick-walled, hyaline. ***Subhymenial hyphae*** hyaline, thin-walled, interwoven or anastomosing, 1–5 μm in diam., occasionally inflated up to 13 μm.

##### Ecology and distribution.

Growing on decayed coniferous wood (*Abies*, *Pinus*, and *Picea* spp.). Currently known only from Sichuan, Xizang, and Yunnan, southwestern China.

##### Specimens examined.

China • Yunnan Province, Deqin County (德钦县), Yunling Town (云岭乡), 28°23'40"N, 098°48'23"E, elev. 3,255 m, 14 August 2016, leg. Y. Li (HMAS277542); • Ninglang County (宁蒗县), en route from Gewa Village (格瓦村) to Labo Town (拉伯乡), 27°43'25"N, 100°31'56"E, elev. 3,440 m, on decaying *Picea* wood, 1 October 2023, leg. T.J. Yu (MHKMU YTJ 185); • Shangri-la City (香格里拉市), near Yijia Village (彝族村), 27°23'11"N, 100°06'30"E, elev. 3,180 m, from decaying *Pinus* wood, 26 August 2020, leg. X. Na (MHKMU NX 221); • ibid., near Yijia Village (彝族村), on decaying *Pinus* wood, 29 July 2021, leg. W.H Zhang (MHKMU ZWH 647); • ibid., near Yijia Village (彝族村), 27°23'14"N, 100°06'31"E, elev. 3,170 m, on decaying *Pinus* wood, 26 August 2020, leg. L.P Tang (MHKMU TLP 3439).

##### Comments.

*Pseudohydnum sinogelatinosum* is one of the most common representatives of the genus in subalpine regions of the Himalayas, where it occurs on decayed wood of *Abies*, *Picea*, and *Pinus* (Zhou et al. 2022; Wang et al. 2025). Previous reports suggested that this species has a predominantly pale-colored pileus. In contrast, our observations reveal an unexpectedly high degree of morphological variability: pileus color ranges extensively, from white, grayish white, pale brown, brown, dark brown, cinnamon, to clay-pink, and the pileus surface varies from smooth to densely covered with minute spines. These findings document considerable intraspecific morphological variation in *P. sinogelatinosum* across its geographic range and underscore the necessity of molecular data for accurate species identification when morphology alone is insufficient.

## Discussion

By integrating multi-marker phylogenetic analyses (ITS–nrLSU–*rpb1*) with detailed morphological reassessment, this study substantially expands our knowledge of *Pseudohydnum* diversity in China. We describe three new species, document the first occurrence of *Pseudohydnum* on bamboo, provide new distributional and morphological data for *P. fusiforme* and *P. laricicola*, and confirm the wide occurrence of *P. sinogelatinosum* in subalpine southwestern China. Two additional lineages (*Pseudohydnum* sp. 1 and sp. 2) remain informally designated due to limited material, suggesting that hidden diversity in the genus may be considerably higher than currently recognized.

### Phylogenetic and biogeographic implications

Our phylogenetic analyses resolve Chinese species into multiple lineages with distinct geographic and ecological affiliations. *Pseudohydnum ellipsosporum* is sister to the temperate northeastern Chinese *P. sinobisporum*, yet the two differ markedly in spore shape and basidial spore number, implying that latitudinal climatic differentiation may have driven allopatric divergence. Similarly, *P. macroumbrosum* forms a sister lineage to the Far Eastern *P. umbrosum*, but possesses strikingly larger basidiomata and more globose spores, suggesting geographic isolation-promoted morphological divergence. *Pseudohydnum roseolostipitatum*, closely related to the Qinghai-Xizang Plateau-endemic *P. himalayanum*, is clearly separated by stipe development and pileus color, this divergence may be plausibly explained by niche differentiation, although this hypothesis requires further ecological testing. These patterns collectively reinforce that morphology alone is, for now, insufficient for species delimitation in this genus.

### Intraspecific morphological variation and its taxonomic implications

A striking finding of this study is the widespread, historically underestimated intraspecific morphological variation within *Pseudohydnum*. In *P. sinogelatinosum*, pileus color varies continuously from white to dark brown and surface texture from smooth to densely strigose, essentially encompassing the macro-morphological range of nearly half the known species. Comparable variation was observed in *P. fusiforme* (subsessile to laterally stipitate basidiomata and wider and more globose basidiospores spores), and *P. laricicola* (larger spores), the latter possibly attributable to the original description having been based on immature specimens. These systematic variations suggest that many traditional diagnostic traits exhibit environmentally induced morphological variation, potentially influenced by developmental stage, micro-environmental factors, or host type. Notably, stipe reduction recurs in multiple independent lineages, offering a valuable opportunity for future ancestral-state reconstruction and phenotype-environment association analyses.

## Conclusions

This study adds three phylogenetically well-supported species to the Chinese *Pseudohydnum* inventory and empirically demonstrates the astonishing extent of intraspecific morphological variation in the genus. Our results confirm that integrating multi-marker molecular data with careful morphological observation is the gold standard for species delimitation in this genus. Continued exploration of the Qinghai-Xizang Plateau and other biodiversity hotspots, combined with genomic and ecological approaches, will likely uncover an even more intricate evolutionary network. This work also highlights the strategic importance of southwestern China in global fungal diversity conservation and calls for systematic inventorying and long-term monitoring of fungal resources in this region.

## Supplementary Material

XML Treatment for Pseudohydnum brunneiceps

XML Treatment for Pseudohydnum ellipsosporum

XML Treatment for Pseudohydnum fusiforme

XML Treatment for Pseudohydnum laricicola

XML Treatment for Pseudohydnum macroumbrosum

XML Treatment for Pseudohydnum roseolostipitatum

XML Treatment for Pseudohydnum sinogelatinosum

## Additional information

### Artificial Intelligence (AI) use

The authors accept full responsibility for the content of the manuscript, including the disclosure of any use of AI.

No AI tools were used in the preparation of this manuscript.

### Author ORCIDs

T.-J. Yu https://orcid.org/0009-0003-6944-7329

W.-H. Zhang https://orcid.org/0000-0002-8983-7346

X.-J. Li https://orcid.org/0009-0004-4591-2940

Y.-Y. Xiong https://orcid.org/0009-0004-7905-3830

Y.-X. Gao https://orcid.org/0009-0007-2276-6831

T. Li https://orcid.org/0009-0004-5873-8371

R. Xue https://orcid.org/0000-0002-9779-0920

Y.-T. Lu https://orcid.org/0000-0002-1278-5584

C. Xu https://orcid.org/0000-0003-0418-5463

J. Li https://orcid.org/0009-0007-2442-7008

L.-P. Tang https://orcid.org/0000-0002-6905-3157

### Data availability

All data that support the findings of this study are available on GenBank, on figshare (https://doi.org/10.6084/m9.figshare.33684880), and in the main text.

## Supplementary materials

Supplementary material 1 Supplementary information Data type xls https://binary.pensoft.net/file/1822776This dataset is made available under the Open Database License (http://opendatacommons.org/licenses/odbl/1.0/). The Open Database License (ODbL) is a license agreement intended to allow users to freely share, modify, and use this Dataset while maintaining this same freedom for others, provided that the original source and author(s) are credited. Tai-Jie Yu, Wen-Hao Zhang, Xiu-Juan Li, Yuan-Yuan Xiong, Yu-Xian Gao, Ting Li, Rou Xue, Yuan-Tian Lu, Chang Xu, Jia Li, Li-Ping Tang
